# Supplementary material for: Optical conversion of pure spin currents in hybrid molecular devices
Source: Nat Commun. 2017 Oct 13;8:926. doi: 10.1038/s41467-017-01034-0 (PMC5640639; doi:10.1038/s41467-017-01034-0)
Supplement: Supplementary file 1 — Supplementary Information [file 41467_2017_1034_MOESM1_ESM.pdf]

**Supplementary Information**

## Supplementary Note 1: Plasmon conversion

Onsager's reciprocity principle relates the off-diagonal responses between fluxes and forces. In our system, one can consider the spin current $I_s$ and the rate of change of the plasmon occupation $dn_\mathrm{p}/dt$ as the fluxes of the phenomena. The associated forces would be the effective magnetic field $H_\mathrm{eff}$ and the radiation pressure $P_\mathrm{r}$. In the absence of dissipation:

$$I_\mathrm{s} = X_\mathrm{ss}\, H_\mathrm{eff} + X_\mathrm{sp} P_\mathrm{r} \tag{1}$$

$$\frac{dn_\mathrm{p}}{dt} = X_\mathrm{ps} H_\mathrm{eff} + X_\mathrm{pp} P_\mathrm{r} \tag{2}$$

with $X_\mathrm{i,j}$ (i,j = s,p) the matrix components of the magnon-plasmon interaction. Onsager reciprocity relations will relate the off-diagonal components, e.g. $X_\mathrm{ps} = -X_\mathrm{sp}$, where the minus sign is because $P_\mathrm{r}$ is even under time-reversal symmetry while $H_\mathrm{eff}$ is odd. In the absence of a driving effective magnetic field,

$$I_\mathrm{s} = X_\mathrm{sp} P_\mathrm{r} \tag{3}$$

so that the light can generate a spin-current. On the other hand, in the absence of light (no radiation pressure), an effective magnetic field driving the magnetization causes a change in the plasmon population:

$$\frac{dn_\mathrm{p}}{dt} = X_\mathrm{ps} H_\mathrm{eff} \tag{4}$$

## Supplementary Discussion

The Gilbert damping $\alpha$ of each system can be compared to the spectroscopic changes observed in that system during FMR. The former can be modulated by changing the ferromagnet under study or its thickness $t$. The spin mixing conductance is proportional to $t$, and large changes in the ISHE induced by a spin current can be measured when making the ferromagnet thicker or thinner. Furthermore, in the case of molecular layers and permalloy, the magnetic coupling can be highly dependent on the electron transfer from the metallic ferromagnet. If using a ferrimagnetic insulator such as yttrium iron garnet ($Y_3Fe_5O_{12}$ or YIG), this coupling is limited to the more indirect exchange interaction.

| | **ΔPLmax [%]**[1] | **Δ$\alpha$ [%]**[2] | **Δ$M_\mathrm{eff}$ [%]**[3] |
|---|---|---|---|
| **Py(30)/**$C_{60}$ | 100 | 50 (0.0135) | -5 |
| **Py(30)/**$Al_2O_3$**/**$C_{60}$ | 12 | 20 (0.0105) | -3 |
| **Py(20)/**$C_{60}$ | 8 | 20 (0.0115) | -9 |
| **PyGd(30)/**$C_{60}$ | 6 | 15 (0.0140) | -22 |
| **YIG/**$C_{60}$**/Al** | 4 | N/A* | -1 |
| **YIG/**$C_{60}$ | -4 | N/A* | -1 |

**Supplementary Table 1|** Resume of the changes in the magnetic parameters and the photoluminescence (PL) at 1.69 eV comparing the signal on/off FMR and on/off the ferromagnetic track. [1]$C_{60}$ photoluminescence enhancement during FMR; [2]Change in the Gilbert damping $\alpha$ with $C_{60}$ deposition, with absolute values in brackets; [3]Change in the effective magnetisation $M_\mathrm{eff}$ with $C_{60}$ deposition.*Changes in the Gilbert damping of YIG films could not be determined due to inhomogeneities and the appearance of multiple resonant peaks after the deposition of $C_{60}$.

The Supplementary Table 1 above gives a resume of the systems measured; larger effects are observed when the Gilbert damping $\alpha$ (associated to the generation and propagation of spin currents) is large. In the case of $C_{60}$ on top of YIG, the changes to the luminescence (and the effective magnetisation) are negligible. Given the lack of conduction electrons and the positive dielectric constant of the insulator and the molecular layer (~15 and 5, respectively), no surface plasmons can be generated. If the $C_{60}$ deposited on YIG is capped with a metallic layer, there is a small but measurable effect in the PL during FMR –which could be attributed to the propagation of part of the spin current through the whole $C_{60}$ layer and the formation of surface plasmons at the $C_{60}$/Al interface. Examples of changes in the FMR peak and line broadening with $C_{60}$ are shown in Supplementary Fig. 1.

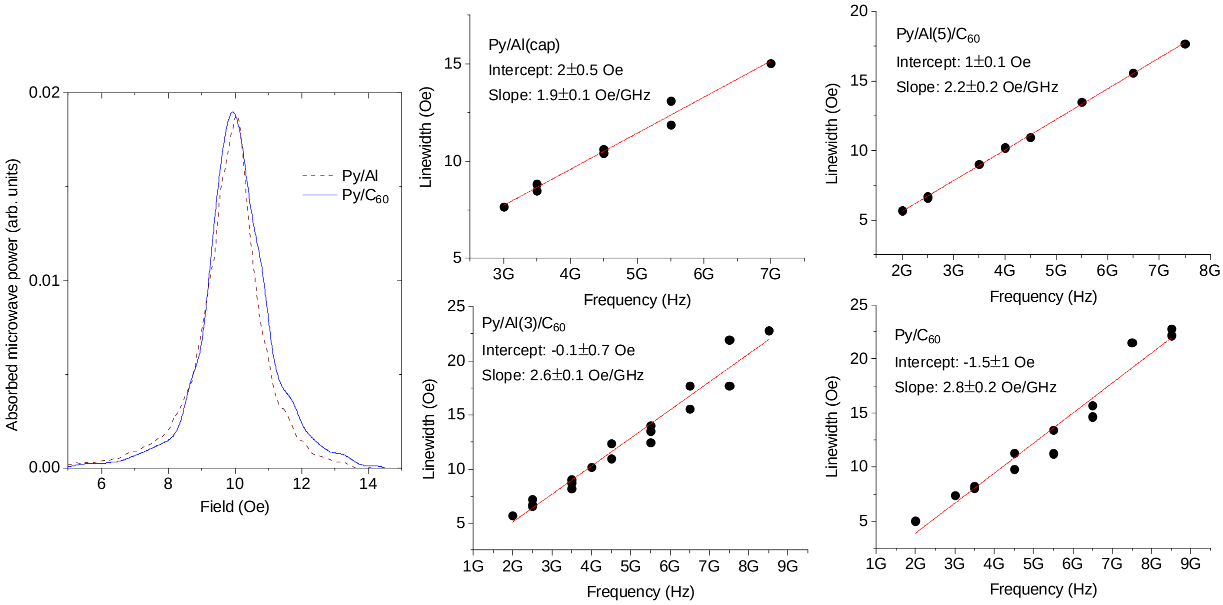


**Supplementary Figure 1|** Data corresponding to the FMR measurements in figure 1. Left: comparison of the resonant peak for a Py film (capped with Al) and a Py/C60 bilaye –note the broadening of the peak. Right: observed resonance linewidths for the different samples leading to the Gilbert damping observed.

The spectroscopy measurements must be taken using low illumination parameters in order to avoid damage to the sample –arising mostly in the form of heating and polymerisation. This implies that each absorption, photoluminescence or Raman map needs several hours for completion. During the absorption measurements at different frequencies displayed in Figure 2 of the main manuscript, we observed a drift in the reflected intensity (Supplementary Figure 2); i.e. the optical absorption of the molecules decreased with time. This could be caused by two phenomena: photobleaching or heating due to the microwaves. When considering the $C_{60}$ area directly on top of the microwave waveguide, this drift was increased, and it was also larger when the microwave emission was started or the frequency increased, which may point to a microwave induced heating. This could explain as well the small increase in reflected intensity (reduced absorbance) seen for frequencies above 1 GHz in Fig. 2H.

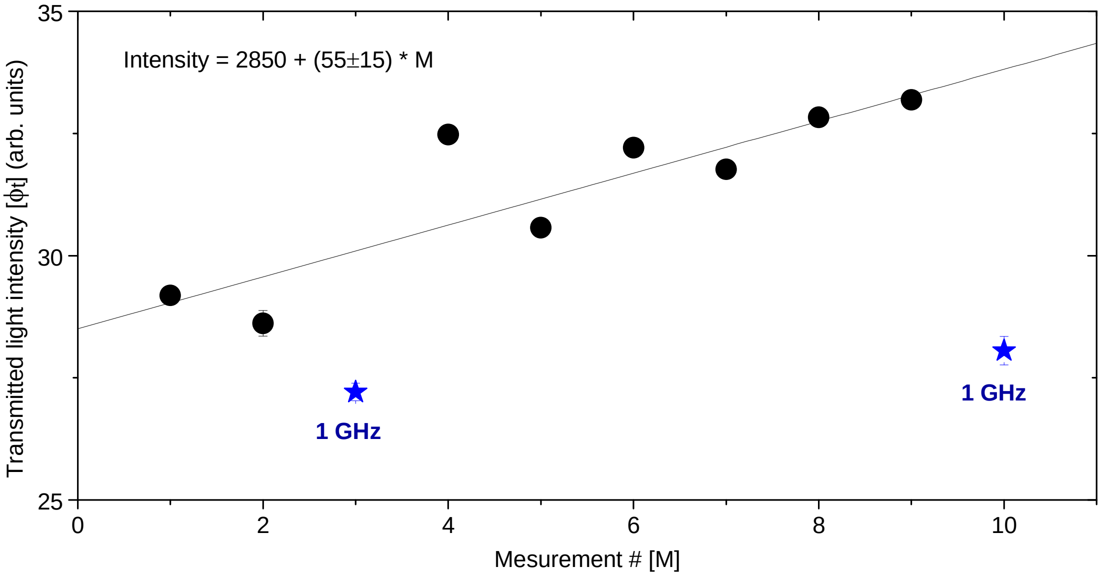


**Supplementary Figure 2|** Changes in the optical transmitivity of the glass/$C_{60}$. There is a linear drift that could be due to heating (of the sample or of the lamp used to illuminate it) and/or photobleaching. Both the drift and the uncertainty in the fit are included in the results displayed in the manuscript. Measurements taken at the resonant frequency of 1 GHz are displayed as blue stars, black dots are measurements at zero or other frequencies. Error bars are the standard error of the mean of the transmitted light averaged over the sample surface.

This bleaching/heating effect is opposite to the reduced reflectivity (increased absorbance) during FMR. The drift and the uncertainty in the linear subtraction (see Supplementary Figure 1) have been taken into account in figure 2 of the main manuscript. Similarly, any heating effect in the PL or Raman spectra would have the reverse effect to that measured during FMR –it should reduce the PL and broaden the Raman spectra. Without making use of this linear correction for the drift, the effect in the optical absorption would be a 21% increase during spin pumping ($\phi_i$ = 47.5 in the units of Fig. 2).

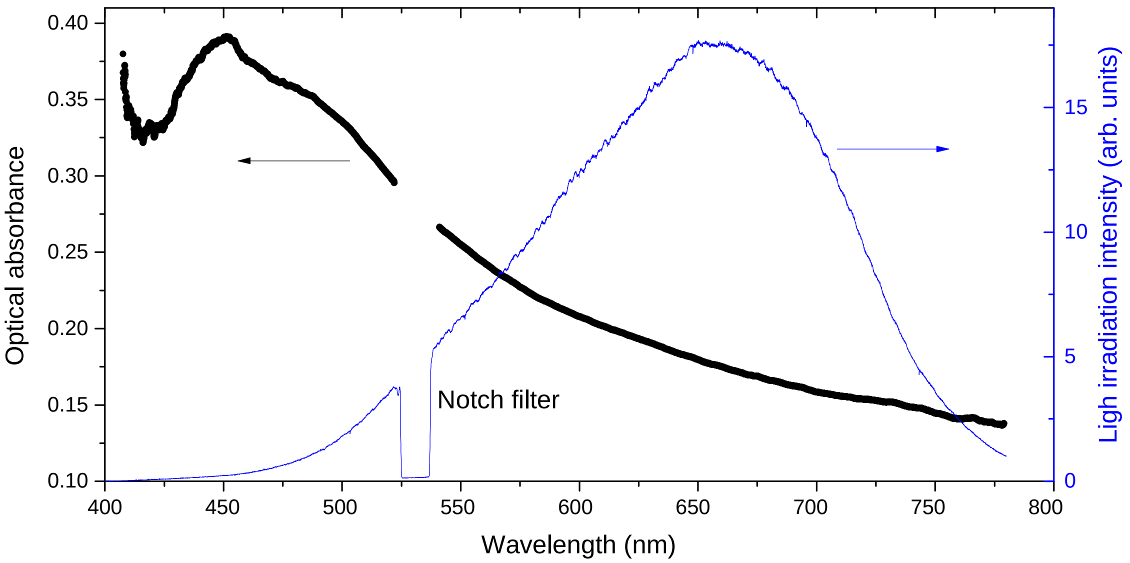


**Supplementary Figure 3|** Optical absorbance of the $C_{60}$ film (black dots) over the visible spectrum and photoemission of the lamp used during our experiments (blue line). The drop in the signal is due to the presence of a notch filter in the system –the optical absorbance in this region cannot be calculated.

The Raman spectra can be fitted to Lorentzian peaks corresponding to the different vibrational modes and replicas. In Supplementary Figure 4 we show the Hg(7)-Ag(2)-Hg(8) region of the spectrum before and during FMR irradiation.The position of the peaks does not change significantly, but the peak width and relative intensities are very different –see Supplementary Table 2 and Fig. 5.

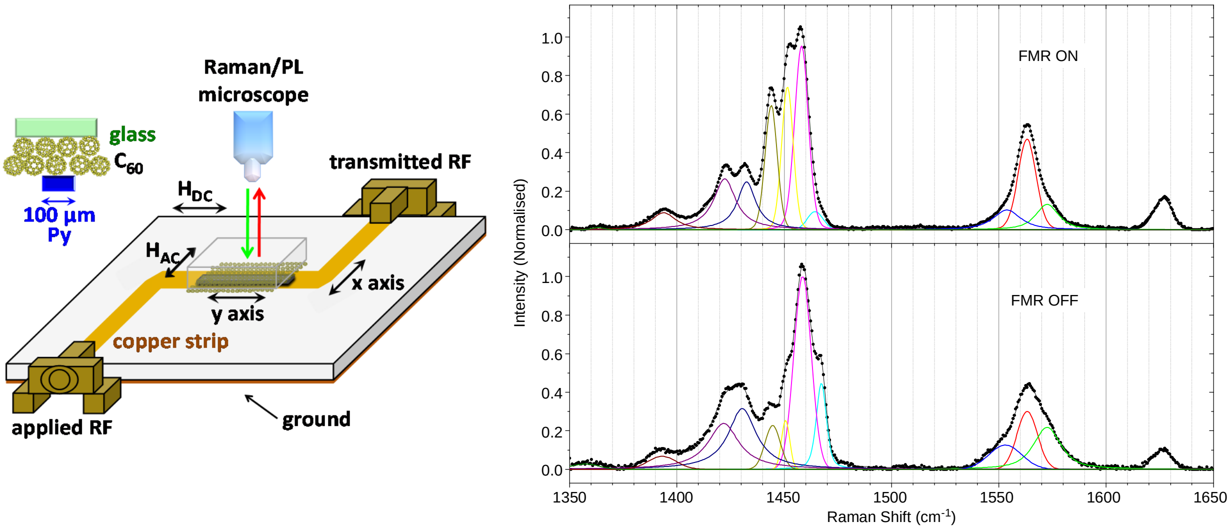


**Supplementary Figure 4|** Left: Schematic of the set up used for FMR-Raman and PL measurements with 100 µm Py or $Al_2O_3$/Py track deposited on the $C_{60}$. Right: Normalised Raman spectrum of $C_{60}$ on Py on and off FMR.

| Mode | Position [cm$^{-1}$] | Width [cm$^{-1}$] (FMR) | Change (%)$^{§}$ | Normalized Amplitude (FMR) | Change (%)$^{§}$ |
|---|---|---|---|---|---|
| Unassigned | 1393 | 15 (→ 14) | NS | 0.07 (→0.09) | NS |
| Hg(7)-T1u(4) | 1423 | 19 (→ 13.5) | -29 | 0.34 (→0.32) | NS |
| | 1431 | 10.5 (→ 7.5) | -29 | 0.23 (→0.2) | NS |
| Ag(2) | 1444 [***] | 10 (→ 7.5) | -25 | 0.25 (→0.69) | 183 |
| | 1451 [**] | 4.5 (→ 5.5) | NS | 0.23 (→0.65) | 189 |
| | 1459 [*] | 10 (→ 8.5) | -15 | 1 | N/A |
| | 1467 | 5.5 (→ 5.5) | NS | 0.45 (→0.06) | -86 |
| Hg(8) | 1553 [**] | 18 (→ 10) | -44 | 0.13 (→0.03) | -73 |
| | 1563 | 11.5 (→ 12) | NS | 0.3 (→0.55) | 81 |
| | 1573 [*] | 15 (→ 9) | -40 | 0.22 (→0.06) | -72 |
| Unassigned | 1626 | 11(→ 10) | NS | 0.10 (→0.16) | NS |

**Supplementary Table 2|** Observed chagnes in the Raman spectrum of $C_{60}$ in contact with Py during FMR –signal normalised to the maximum peak at 1459 cm$^{-1}$. The vibrational peaks become narrower, an effect seen in some SERS measurementS The intensity of the Ag(2)*** and Ag(2)** replicas is greatly enhanced during FMR, whereas the Ag(2) is quenched. The main Hg(8) mode is also enhanced, but the Hg(8)** and Hg(8)* replicas are reduced so the total area for the Hg(7-8) modes is reduced. $^{§}$Changes ≤1 cm$^{-1}$ in width or ≤0.1 in amplitude are considered not significant (NS).

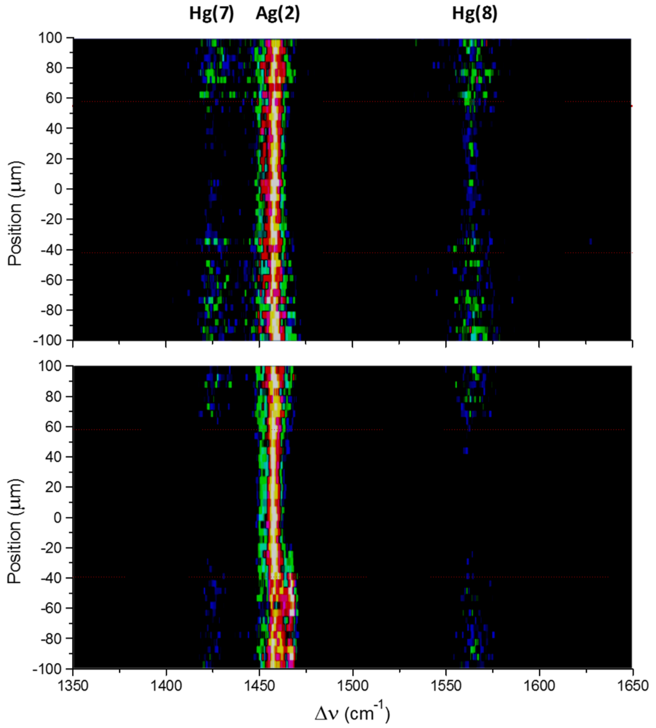


**Supplementary Figure 5|** Raman spectrum for $C_{60}$ deposited on a $Al_2O_3$/Py track (as results for Supplementary Figure 2 and Table 2 but with an insulating barrier between the ferromagnet and the fullerenes) before (top) and during (bottom) FMR. In the region of the Py track (delimited by the horizontal red lines), it is possible to observe a red shift of the Ag(2) peak and a relative drop of the intensity of the Hg modes.

The PL spectrum of $C_{60}$ is an ideal tool in terms of potential applications and for characterisation of the changes in the molecular film. The standard analysis of the $C_{60}$ PL spectra is displayed in Supplementary Figure 6.

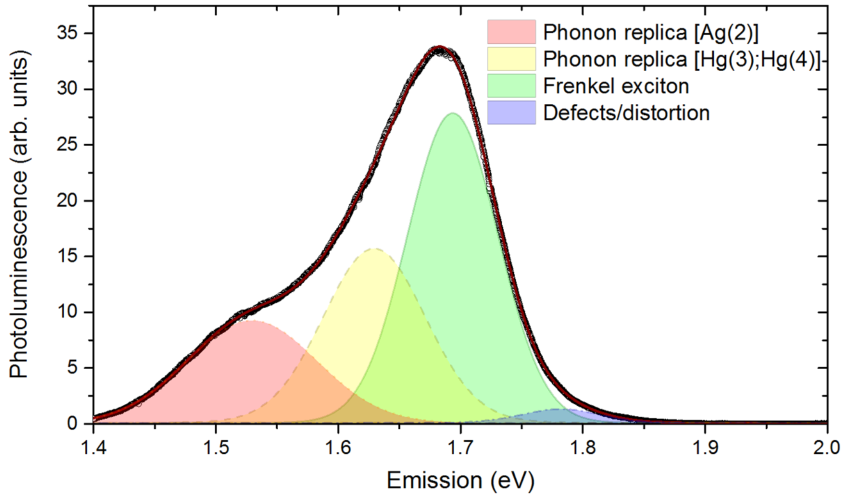


**Supplementary Figure 6|** Typical PL signal from a $C_{60}$ film deposited on Py. The spectrum shows the main Frenkel exciton at 1.69 eV corresponding to the HOMO-LUMO transition, which can be mediated by the Ag(2) and Hg(3)-Hg(4) vibrational modes at 1470 and 710-750 $cm^{-1}$ (~0.18 and 0.09 eV), respectively. These vibron-mediated transitions give rise to phonon replicas at 1.6 and 1.51 eV. There is a small peak at ~1.77 eV that is commonly associated to defects, distortions in the lattice and/or oxidation.

The changes in photoluminescence during FMR are localised to the $C_{60}$ region in contact with, or close to, the ferromagnet. This is the case both for $C_{60}$ directly connected with permalloy and when an insulating barrier is placed in between. However, for the latter, the effect is smaller and decays away from the track –see Supplementary Figures 6 and 7.

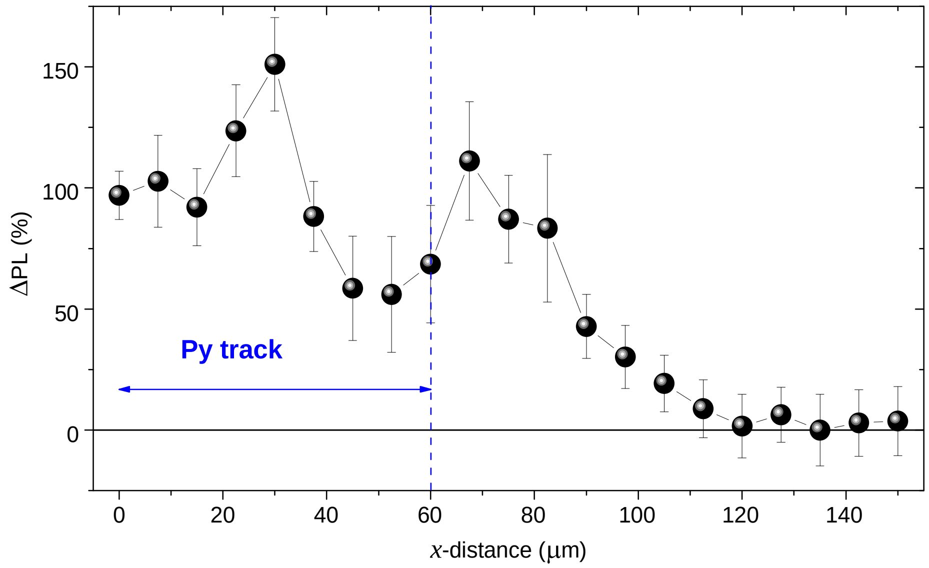


**Supplementary Figure 7|** Dependence of the photoluminescence change $\Delta PL$ during FMR with the distance to the centre of the spin pumping Py track. The effect drops away from the ferromagnet, but it is still possible to measure a change in PL for some ~40 μm away from the nominal track limits. This could be partly because deposition through a shadow mask can result in a smooth boundary and magnetic material extending for some 10s of μm. Also, the gaussian profile of the beam and observation through the glass substrate result in beam refraction and dispersion of the light spot with a probing area of ~10 μm in size. Error bars are the standard error of the mean in the photoluminescence change averaged in the y-direction (parallel to the magnetic track).

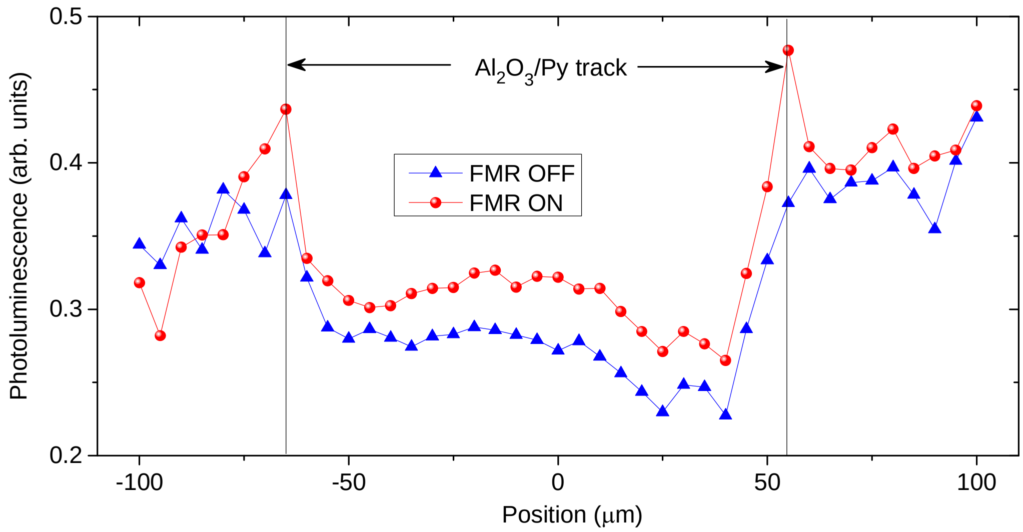


**Supplementary Figure 8|** a. Changes in the PL intensity at 1.65-1.75 eV for $C_{60}$ with a 100 mm wide $Al_2O_3$/Py track deposited on top. During FMR/spin pumping, the PL doesn't change outside the track, whereas there is a ~12% enhancement for $C_{60}$ with $Al_2O_3$/Py on top.

Spin pumping in molecules generated by ferrimagnetic insulators is dominated by the exchange interaction (*34*). Small or nil changes in the spectroscopy are observed when using an insulating ferrimagnet instead of metallic permalloy for spin pumping. This is likely due to the low free carrier density and the continuously positive dielectric constant across the YIG/$C_{60}$ interface, which prevents the formation of surface plasmons –see Supplementary Figures 8 and 9.

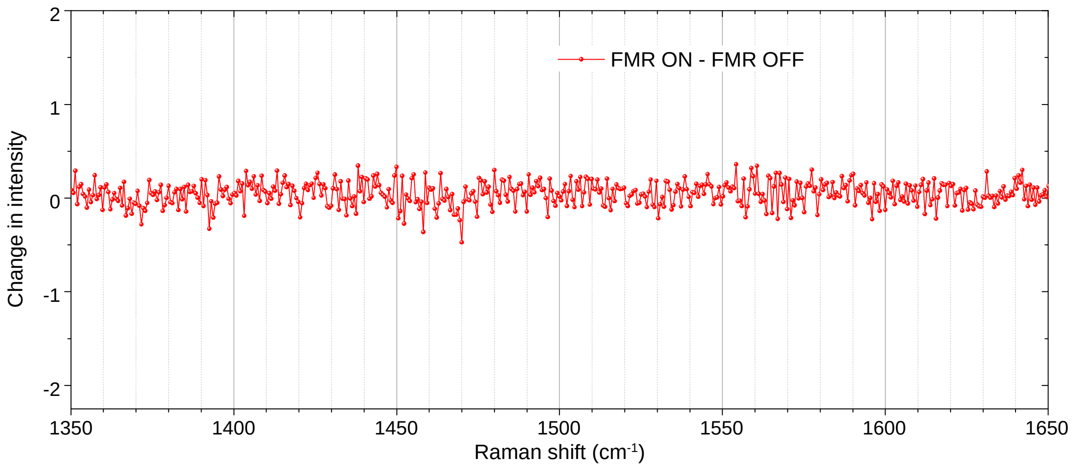


**Supplementary Figure 9|** Contrasting with the measurements on metallic ferromagnets, the Raman spectrum of $C_{60}$ on top of the insulating ferrimagnet yttrium iron garnet (YIG) shows no changes during FMR.

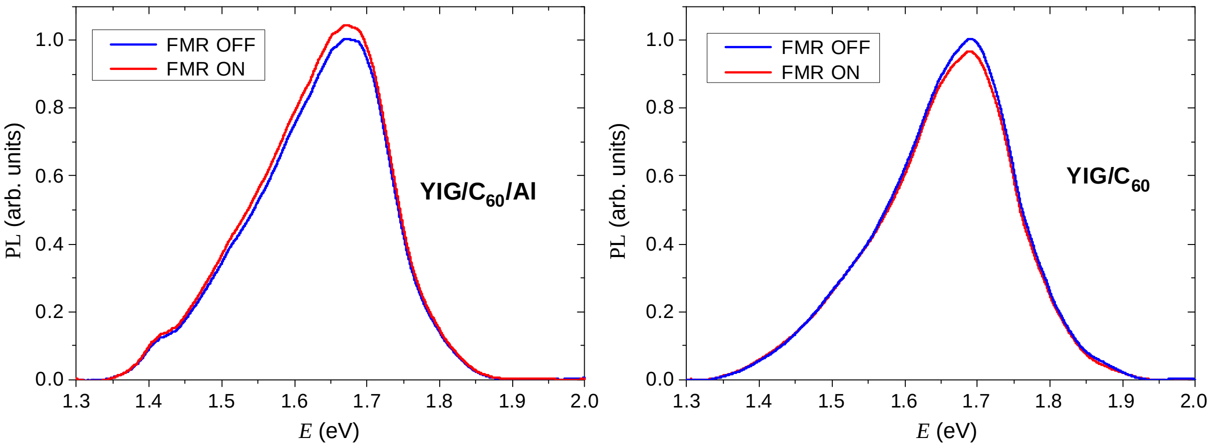


**Supplementary Figure 10|** Changes in the PL of $C_{60}$ deposited on YIG with and without a metallic cap layer. The changes during FMR are small in both cases (0-4% depending on the emission considered). The difference in sign could be due to two competing interactions: enhanced efficiency from induced plasmons (PL increase) vS heating from the microwaves (PL reduction).For Al-capped $C_{60}$, the top metal layer provides the electron cloud needed for the formation of surface plasmons at the interface, although the spin current decays across the molecular layer and therefore the effect is small. In the case of YIG/$C_{60}$, it is not possible to generate plasmons, so the heating effect dominates.

Transport measurements show a change in the conductivity of $C_{60}$ films during FMR. This effect is shown in Supplementary Figure 11 by comparing the I-V characteristic before and during FMR for Py(30)/$Al_2O_3$(2)/Au(10) and Py(30)/$Al_2O_3$(0.9)/$C_{60}$(10)/Au(10) –thickness in brackets in nm. For samples with a single, thick $Al_2O_3$ insulating layer, the transport mechanism is electron tunnelling. These samples show a small change in conductivity during FMR (<0.5%) due to the high energy barrier seen by the carriers and/or the small spin pumping efficiency. However, the change is apparent in devices with a porous barrier and a molecular layer between the electrodes. Here, the alumina spacer stops the hybridization of the metallo-molecular interface but does not completely prevent spin pumping into the buckyballs. The change in conductivity is asymmetric, with larger changes when the electrons flow from the ferromagnet. The origin of the effect could be the formation of a weakly magnetised or spin polarised region at the metallo-molecular interface due to the induced spin imbalance. This in turn would favour the injection of majority electrons from the ferromagnet, resulting in a change of the resistance during spin pumping. These changes could also be caused by local heating or phonons in the ferromagnet, thus improving the hopping probability in the molecular film. However, given the low magnetoelastic coupling of Py, phonon generation should be a very minor mechanism of power dissipation –see below.

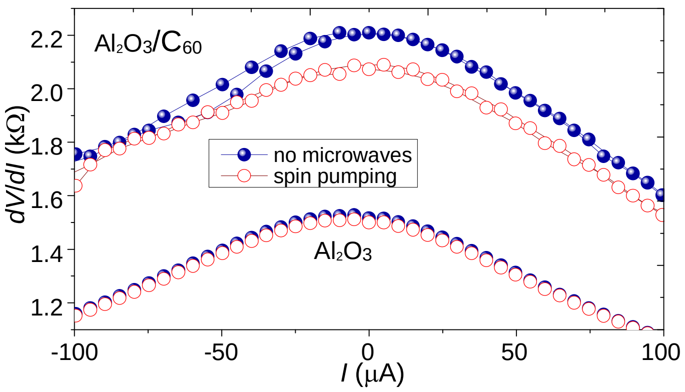


**Supplementary Figure 11|** Resistance vs. current bias through Py(30)/$Al_2O_3$(0.9)/$C_{60}$(10)/Au(10) and Py(30)/$Al_2O_3$(2)/Au(10) junctions. The resistance change during spin pumping is only apparent in the sample with $C_{60}$, and larger when the electrons are injected from the Py.
